# High-Temperature Mechanical Characterization of CeO_2_ as a Ceramic Surrogate Fuel Based on FIB and Nanoindentation

**DOI:** 10.3390/ma19102134

**Published:** 2026-05-19

**Authors:** Jiaxuan Si, Jiajun Xu, Shiqiang He, Dongsheng Xie, Changfeng Dong, Pengcheng Zhu, Risheng Qiu

**Affiliations:** 1 The First Sub-Institute, Nuclear Power Institute of China, Chengdu 610041, China; sjx@npic.ac.cn (J.S.); dcf@npic.ac.cn (C.D.);; 2 International Joint Laboratory for Light Alloys (MOE), College of Materials Science and Engineering, Chongqing University, Chongqing 400044, Chinaqjczz211@163.com (S.H.)

**Keywords:** nuclear fuel, ceramic oxide, high-temperature nanoindentation, mechanical properties

## Abstract

CeO_2_ is widely used as a non-radioactive surrogate for UO_2_ because of its fluorite crystal structure and similar thermophysical characteristics. In this study, an FIB-assisted specimen preparation route combined with high-temperature nanoindentation was used to evaluate the micromechanical behavior of CeO_2_ from room temperature to 400 °C. Hardness and Young’s modulus were experimentally measured at room temperature, 100 °C, 200 °C, 300 °C, and 400 °C. The load–displacement curves were smooth, and no obvious pop-in events were observed within the tested load range. From 100 °C to 400 °C, both Young’s modulus and hardness decreased approximately linearly with increasing temperature, and linear fitting was used to describe their temperature dependence. The measured Young’s modulus decreased from 191.3 ± 14.0 GPa at 100 °C to 136.7 ± 9.5 GPa at 400 °C, while the hardness decreased from 6.79 ± 0.58 GPa to 5.08 ± 0.48 GPa. The obtained temperature-dependent trend is consistent with previously reported high-temperature nanoindentation data for fluorite-structured oxides. These results provide useful micromechanical data and methodological support for elevated-temperature small-scale mechanical characterization of ceramic nuclear fuel surrogate materials.

## 1. Introduction

Nuclear energy is an important low-carbon energy source and plays a critical role in energy security and the transition toward cleaner power systems [1]. Uranium dioxide (UO_2_) has long been the dominant fuel material in commercial nuclear reactors because of its high melting point, chemical stability, and compatibility with reactor operating conditions [2]. During service, UO_2_ fuel pellets are subjected to high temperature, mechanical stress, irradiation damage, fission gas accumulation, and microstructural evolution. These factors can significantly affect the local elastic stiffness, hardness, cracking behavior, and deformation response of the fuel [3,4,5,6,7,8]. Such changes are closely related to pellet–cladding mechanical interaction (PCMI), which is one of the key issues influencing fuel performance and safety. Reliable local mechanical property data are therefore necessary for understanding fuel degradation, improving fuel performance models, and supporting the design of advanced nuclear fuel systems.

Direct mechanical characterization of irradiated UO_2_ is technically challenging because spent fuel contains highly radioactive fission products and requires shielded hot-cell facilities [9]. Irradiation campaigns also involve long experimental cycles, including reactor exposure, cooling, and post-irradiation examination [10]. In addition, conventional bulk mechanical testing has limited capability for resolving the heterogeneous local response caused by grain boundaries, pores, irradiation defects, fission products, and local cracking. These challenges have motivated the use of non-radioactive surrogate materials and small-scale mechanical testing methods to obtain local mechanical information under controlled laboratory conditions.

Small-scale mechanical testing provides an effective approach for characterizing local mechanical behavior while requiring only a very small material volume [11,12,13,14]. Among these techniques, nanoindentation is particularly useful for ceramic nuclear fuel and surrogate materials because it can determine hardness and elastic modulus from load–displacement data with high spatial resolution [15,16,17]. When combined with elevated-temperature capability, nanoindentation can further reveal the temperature-dependent micromechanical response of fuel-related ceramics. However, the high-temperature nanoindentation of ceramic micro-specimens remains sensitive to surface condition, local defects, thermal drift, and specimen mounting, making specimen preparation and data interpretation important aspects of the testing procedure [18].

Cerium dioxide (CeO_2_) is a representative fluorite-structured oxide and has been widely used as a non-radioactive surrogate for UO_2_ because the two materials share similar crystal structures, bonding characteristics, and high-temperature stability [19,20,21,22]. CeO_2_ is chemically stable, non-radioactive, and readily fabricated as dense ceramic specimens, making it suitable for methodological development and preliminary micromechanical evaluation. Previous studies have reported room-temperature elastic modulus, hardness, and thermal properties of CeO_2_ [23,24,25], irradiation-induced microstructural evolution in CeO_2_ [19,20,21,22,26], and high-temperature mechanical properties of CeO_2_ and related fluorite-structured oxides [10,27]. Nevertheless, high-temperature micromechanical data for CeO_2_ remain relatively limited compared with room-temperature data, and further experimental information is still needed to understand its local mechanical response over an elevated-temperature range.

In this study, an FIB-assisted specimen preparation route combined with high-temperature nanoindentation was used to characterize the micromechanical behavior of CeO_2_ from room temperature to 400 °C. Young’s modulus and hardness were obtained at room temperature, 100 °C, 200 °C, 300 °C, and 400 °C, and the temperature-dependent trends were compared with the available literature data. EBSD and post-indentation SEM observations were used to examine the microstructure and representative indentation morphologies. The results provide useful local mechanical property data for CeO_2_ and a reference for the elevated-temperature small-scale mechanical characterization of ceramic nuclear fuel surrogate materials.

## 2. Experimental Procedures

### 2.1. Specimen Preparation and Nanoindentation Testing

The material used in this study was bulk CeO_2_ ceramic sintered at 1200 °C. The density of the sintered CeO_2_ ceramic was measured by the Archimedes drainage method to be 6.88 g cm^−3^. Taking the theoretical density of CeO_2_ as 7.22 g cm^−3^, the relative density was calculated to be approximately 95.3%. To mimic the preparation route for small-volume ceramic fuel specimens, the bulk specimen was sectioned, ground, and mechanically pre-polished using a Leica-integrated grinding and polishing system. Final surface finishing was then carried out by argon-ion polishing to obtain a smooth surface suitable for FIB machining and nanoindentation.

**Figure 1:**
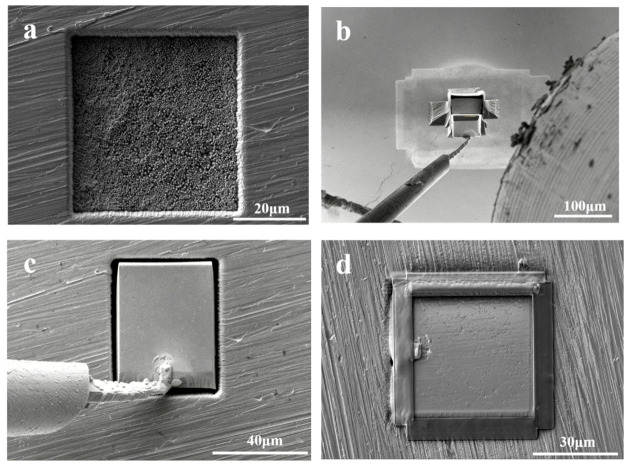
FIB-assisted preparation and mounting of the CeO_2_ micro-specimen: (**a**) milling of a recess in the 316 L holder; (**b**) extraction of the CeO_2_ micro-specimen; (**c**) transfer of the specimen into the 316 L recess; (**d**) final specimen after Pt deposition.

A 316 L cylindrical holder with a diameter of 5 mm and a height of 1.2 mm was first prepared, and a cavity measuring 42.0 μm × 42.0 μm × 11.0 μm was milled into its surface by focused ion beam (FIB), as shown in Figure 1a. This cavity served as the receiving pocket for the CeO_2_ micro-specimen. A CeO_2_ block with dimensions of approximately 40 μm × 40 μm × 11.3 μm was then cut from the bulk sample by FIB (Figure 1b). During rough machining, a relatively high ion-beam current was used to remove material efficiently and isolate a central micro-cube. The beam current was subsequently reduced for fine cutting of the sidewalls, and a final low-current polishing step was applied from multiple directions to minimize the damaged layer and obtain a flat, clean surface.

After preparation of the 316 L holder, the EasyLift manipulator (Thermo Fisher Scientific, Waltham, MA, USA) was attached to the specimen by Pt deposition, allowing the specimen to be separated from the bulk and positioned accurately in the cavity, as illustrated in Figure 1c. Mounting the CeO_2_ specimen in the 316 L pocket facilitated safe transfer and provided a mechanically stable configuration for subsequent nanoindentation.

Once the specimen had been positioned in the cavity, Pt was locally deposited to fill the gap between the specimen and the cavity walls and thereby improve bonding strength. A second Pt deposition step was then applied around the peripheral region of the top surface. This treatment was intended to improve electrical conductivity, suppress charge accumulation during microscopy and indentation, and provide additional surface protection during elevated-temperature testing. The final mounted specimen is shown in Figure 1d.

Before nanoindentation, the mounted specimen was characterized by electron backscatter diffraction (EBSD) in an FIB-SEM dual-beam system equipped with an EBSD detector to evaluate the crystallographic orientation distribution of the tested region.

After EBSD characterization, the 316 L holder carrying the CeO_2_ specimen was mounted in a Hysitron TI-980 nanoindenter (Bruker, Minneapolis, MN, USA). Quasi-static nanoindentation tests were conducted at room temperature, 100 °C, 200 °C, 300 °C, and 400 °C using a Berkovich indenter and a maximum load of 5000 μN. Before each test, the specimen was held at the target temperature for 300 s to ensure thermal stabilization. Each loading cycle consisted of loading to 5000 μN in 4 s, holding at the peak load for 2 s, and unloading in 5 s. Five indentation tests were performed at each temperature.

### 2.2. Data Analysis

Nanoindentation load–displacement curves were analyzed using the Oliver–Pharr method [15]. For each temperature, Young’s modulus and hardness were reported as the average value ± standard deviation based on five indentation measurements. The contact stiffness, S, was obtained from the initial unloading segment of the load–displacement curve:

(1) $$S=d_{p}/d_{h}$$

After obtaining contact stiffness, the contact depth ($h_{c}$) and contact area (*A*) can be calculated as follows:

(2) $$h_{c}=h_{\max}-\varepsilon \frac{P_{\max}}{S}$$

In Equation (2), *h*_max_ is the maximum displacement, *P*_max_ is the maximum load, and *ε* is a constant related to the indenter shape (*ε* = 0.75 for a Berkovich indenter). For an ideal Berkovich indenter, the contact area *A* can be expressed as

(3) $$A=24.56{h_{c}}^{2}$$

Once the contact area *A* is obtained, the hardness and modulus of the specimen can be calculated as follows:

(4) $$H=\frac{P_{\max}}{A}$$

(5) $$\frac{1}{E_{r}}=\frac{1-\nu^{2}}{E}+\frac{1-{\nu_{i}}^{2}}{E_{i}}$$

In Equation (5), *E* and *v* are the elastic modulus and Poisson’s ratio of the tested material, respectively; *E_i_* and *v_i_* are the elastic modulus and Poisson’s ratio of the indenter, respectively. For a diamond indenter, *E_i_* = 1141 GPa and *v_i_* = 0.07. The reduced modulus *E_r_* can be obtained by the following formula:

(6) $$E_{r}=\frac{S\sqrt{\pi }}{2\beta \sqrt{A}}$$

In Equation (6), *β* is a constant related to the geometric shape of the indenter, and *β* = 1.034 is generally adopted for a Berkovich indenter [15].

## 3. Results

**Figure 2:**
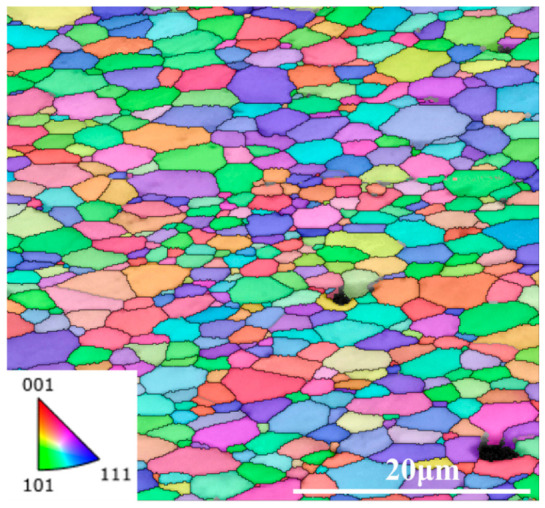
EBSD inverse pole figure map of the CeO_2_ specimen.

Figure 2 shows the EBSD inverse pole figure (IPF) map of the CeO_2_ specimen. The indexed area is consistent with the fluorite CeO_2_ phase, and the IPF map shows a polycrystalline microstructure without obvious preferred orientation. Most grains were several micrometers to more than ten micrometers in size, and the overall grain distribution was relatively uniform. Several dark regions are visible in the EBSD map, suggesting the presence of pores or locally unindexed areas in the tested region.

**Figure 3:**
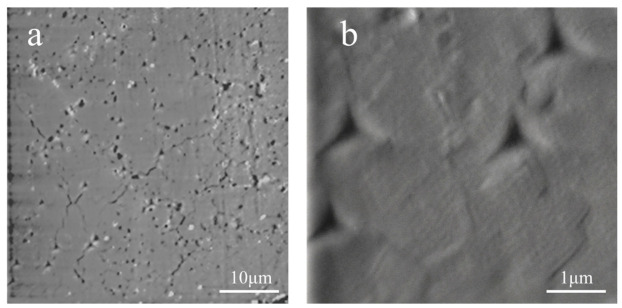
SEM images of indentation impressions after nanoindentation: (**a**) low-magnification image showing multiple indentation impressions and local radial cracks; (**b**) enlarged representative indentation impressions without obvious crack extension.

SEM images of indentation impressions after nanoindentation are shown in Figure 3. The post-indentation morphologies show heterogeneous cracking behavior in the CeO_2_ specimen. Some indentation impressions exhibited radial cracks, whereas others showed no obvious crack extension. This difference suggests that indentation-induced cracking is sensitive to local microstructural heterogeneity, including the pores observed in the EBSD map and local variations in the region beneath the indenter.

**Figure 4:**
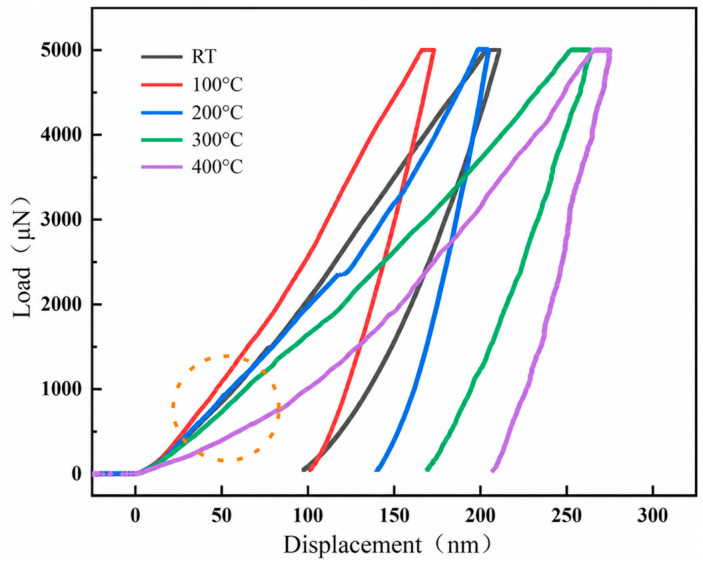
Nanoindentation load–displacement (P–h) curves of CeO_2_ at different temperatures. The curves are distinguished by color: RT, black; 100 °C, red; 200 °C, blue; 300 °C, green; and 400 °C, purple. The orange dashed circle highlights the low-load region where the curves begin to separate.

Figure 4 shows the nanoindentation load–displacement (P–h) curves obtained from room temperature to 400 °C. For all five temperatures, the loading curves were continuous and smooth, with no obvious pop-in events or displacement bursts [28]. This indicates that the elastic-to-plastic transition remained gradual within the tested load range. During the initial loading stage, the 100 °C curve exhibited the steepest slope, corresponding to the highest apparent stiffness. The slopes at room temperature and 200 °C were similar, and the overall trend from 100 °C to 400 °C showed a decrease in slope with increasing temperature, indicating a reduction in elastic modulus [29].

Additionally, it can be observed that the loads required to achieve the same displacement varied significantly at different temperatures, and the maximum displacements generated under the same maximum load also differed greatly across temperatures. At 400 °C, the material only required a load of less than 1000 μN to produce a displacement of approximately 100 nm, whereas at 100 °C (where hardness and modulus were the highest), a load of nearly 3000 μN was needed. The maximum displacement at 400 °C could reach up to 200 nm, while it was only about 100 nm at room temperature and 100 °C.

The average Young’s modulus and hardness values obtained from nanoindentation are listed in Table 1. The values are reported as the mean ± standard deviation from five indentation measurements at each temperature. Room-temperature Young’s modulus and hardness were 167.6 ± 12.5 GPa and 6.44 ± 0.72 GPa, respectively. At 100 °C, the Young’s modulus and hardness increased to 191.3 ± 14.0 GPa and 6.79 ± 0.58 GPa, respectively. From 100 °C to 400 °C, both Young’s modulus and hardness decreased progressively with increasing temperature. The Young’s modulus decreased from 191.3 ± 14.0 GPa at 100 °C to 136.7 ± 9.5 GPa at 400 °C, while the hardness decreased from 6.79 ± 0.58 GPa to 5.08 ± 0.48 GPa over the same temperature range.

## 4. Discussion

The load–displacement curves and post-indentation morphologies indicate that the mechanical response of CeO_2_ under nanoindentation is governed by both temperature-dependent softening and local microstructural heterogeneity. CeO_2_ has a cubic fluorite structure with strong ionic bonding. Although the cation sublattice is face-centered cubic, dislocation activity in fluorite-structured oxides is generally limited by bonding character and the resistance to slip-system activation [20]. Therefore, CeO_2_ tends to exhibit limited plastic accommodation and brittle deformation under indentation, especially at relatively low temperatures. The absence of obvious pop-in events in the present load–displacement curves suggests that abrupt displacement bursts or sudden dislocation nucleation events were not dominant within the tested load range. Instead, the deformation proceeded in a relatively continuous manner, accompanied in some locations by indentation-induced radial cracking.

**Figure 5:**
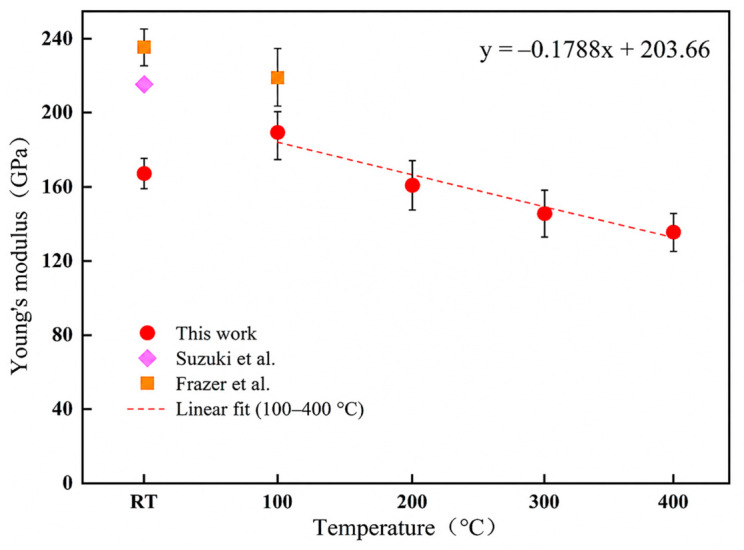
Temperature dependence of Young’s modulus for CeO_2_. Red circles represent the results from this work, the purple diamond represents the data reported by Suzuki et al. [23], and orange squares represent the data reported by Frazer et al. [10]. The red dashed line represents the linear fit of the Young’s modulus data from 100 °C to 400 °C.

Figure 5 shows the variation in Young’s modulus with temperature. To describe temperature dependence, Young’s modulus data from 100 °C to 400 °C were fitted linearly, and the fitted relationship is given by Equation (7):

(7) E = −0.1788T + 203.66

where E is Young’s modulus in GPa and T is the temperature in °C. The experimental room-temperature value is shown in Figure 5 and Table 1, but the linear fitting was performed using the data from 100 °C to 400 °C, where a consistent decreasing trend was observed. The fitted relation gives an extrapolated modulus of approximately 200 GPa at 20 °C. This value is close to the room-temperature values reported by Suzuki et al. [23], particularly the resonant ultrasound spectroscopy result of approximately 207 GPa, and is also reasonably close to the nanoindentation value of approximately 215 GPa. The agreement between the extrapolated value and reported literature values supports the reliability of the measured high-temperature trend, although the absolute modulus obtained by nanoindentation may be influenced by local microstructure, surface condition, and testing volume.

Further comparison with the elevated-temperature nanoindentation data reported by Frazer et al. for CeO_2_ and other fluorite-structured oxides [10], as well as recent results for (U, Ce)O_2_ compounds [27], shows that the present results follow a similar temperature-dependent softening trend. Both studies show a progressive decrease in Young’s modulus with increasing temperature, which is consistent with thermal softening in fluorite-structured ceramic oxides. The modulus values obtained in the present work are slightly lower than some reported literature values at higher temperatures. Such differences are reasonable for nanoindentation measurements because the measured response is highly localized and can be affected by grain orientation, porosity, surface preparation, indentation position, and the specific data-reduction procedure. In the present specimen, the pores or locally unindexed regions observed in the EBSD map may also contribute to the lower measured modulus and to the scatter represented by the standard deviations.

**Figure 6:**
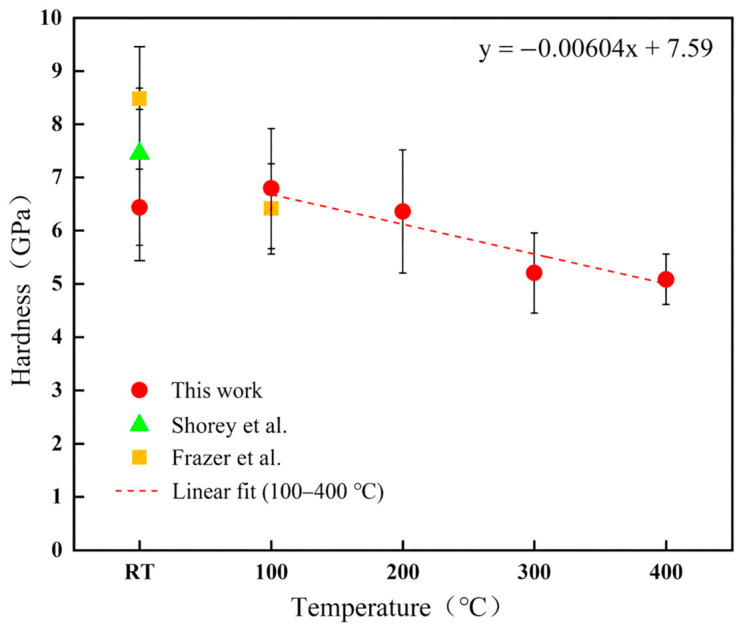
Temperature dependence of hardness for CeO_2_. Red circles represent the results from this work, green triangles represent the literature data reported by Shorey et al. [25], and orange squares represent the literature data reported by Frazer et al. [10]. The red dashed line represents the linear fit of the hardness data from 100 °C to 400 °C.

Figure 6 shows the variation in hardness with temperature. Similarly to Young’s modulus, the hardness data from 100 °C to 400 °C were fitted linearly to further describe the temperature dependence, and the fitted relationship is given by Equation (8):

(8) H = −0.00604T + 7.59

where H is hardness in GPa and T is the temperature in °C. The hardness decreased from 6.79 ± 0.58 GPa at 100 °C to 5.08 ± 0.48 GPa at 400 °C, indicating a clear reduction in resistance to indentation deformation with increasing temperature. Extrapolation of the fitted relation to 20 °C gives an estimated hardness of approximately 7.47 GPa. This value falls within the range of room-temperature nanoindentation hardness values reported in the literature [10,24,25] and is reasonably close to the value reported by Frazer et al. [10]. The decreasing hardness with increasing temperature can be attributed to the thermally assisted deformation of the ceramic lattice and the reduced resistance to localized contact deformation at elevated temperature.

The experimentally measured room-temperature hardness and Young’s modulus are lower than those measured at 100 °C. This behavior should be considered together with the standard deviations and the local nature of nanoindentation. Since each indentation samples only a small volume, the measured values can be affected by surface condition, local pores, grain orientation, and the position of the indentation relative to microstructural features. Therefore, the lower room-temperature values are most likely associated with local testing scatter and near-surface condition rather than a general intrinsic strengthening effect between room temperature and 100 °C. For this reason, the temperature-dependent fitting in Figure 5 and Figure 6 was based on the data from 100 °C to 400 °C, where both Young’s modulus and hardness show a consistent decreasing tendency.

The indentation morphologies provide further information on the local deformation and fracture behavior of the CeO_2_ specimen. As shown in Figure 3, some indentation impressions were accompanied by radial cracks, whereas others showed no obvious crack extension. According to indentation fracture models for brittle ceramics [30], crack initiation and propagation are strongly affected by the local stress field, fracture resistance, and pre-existing microstructural defects. In the present study, the coexistence of cracked and crack-free impressions suggests that the indentation fracture behavior of CeO_2_ is spatially heterogeneous. The dark regions observed in the EBSD map are likely related to pores or locally unindexed areas, which can act as local stress concentrators and promote crack initiation under indentation loading.

Although indentation-induced cracks were observed in some impressions, well-developed and uniformly measurable radial crack systems were not obtained for all indents. Therefore, the present SEM observations are used mainly to discuss the qualitative cracking behavior rather than to calculate fracture toughness. Local residual stresses may also contribute to the variation in crack initiation. Such stresses can be introduced during sintering, FIB processing, Pt deposition, or thermal cycling, and may locally modify the tensile or compressive stress state beneath the indenter. Together, the observed pores, indentation cracking, and data scatter indicate that microstructural heterogeneity should be considered when interpreting nanoindentation-derived mechanical properties of CeO_2_ ceramics.

Overall, the present results show that Young’s modulus and the hardness of CeO_2_ decrease progressively from 100 °C to 400 °C, in agreement with previously reported elevated-temperature nanoindentation data for fluorite-structured oxides. The combination of FIB-assisted specimen preparation, EBSD characterization, post-indentation SEM observation, and high-temperature nanoindentation provides a useful route for evaluating the local micromechanical response of CeO_2_ ceramic surrogate materials.

## 5. Conclusions

A FIB-assisted specimen preparation route combined with high-temperature nanoindentation was applied to characterize the local micromechanical response of CeO_2_ ceramic from room temperature to 400 °C. The EBSD results showed a polycrystalline CeO_2_ microstructure with local pores or unindexed regions, while post-indentation SEM observations revealed locally variable indentation-induced cracking behavior. No obvious pop-in events were observed in the load–displacement curves within the tested load range, indicating a relatively continuous indentation response under the present testing conditions.

Young’s modulus and hardness were experimentally measured at room temperature, 100 °C, 200 °C, 300 °C, and 400 °C. From 100 °C to 400 °C, both Young’s modulus and hardness decreased progressively with increasing temperature. Young’s modulus decreased from 191.3 ± 14.0 GPa at 100 °C to 136.7 ± 9.5 GPa at 400 °C, while hardness decreased from 6.79 ± 0.58 GPa to 5.08 ± 0.48 GPa. The temperature-dependent trends were consistent with previously reported high-temperature nanoindentation data for CeO_2_ and related fluorite-structured oxides. The results provide useful local mechanical property data for CeO_2_ and support the use of FIB-assisted high-temperature nanoindentation for the small-scale mechanical characterization of ceramic nuclear fuel surrogate materials.

## Figures and Tables

**Table 1:** Average Young’s modulus and hardness of the CeO_2_ specimen measured by nanoindentation at different temperatures.

Temperature (°C)	Young’s Modulus (GPa)	Hardness (GPa)
RT	167.6 ± 12.5	6.44 ± 0.72
100	191.3 ± 14.0	6.79 ± 0.58
200	161.4 ± 13.2	6.36 ± 0.84
300	146.4 ± 10.8	5.21 ± 0.62
400	136.7 ± 9.5	5.08 ± 0.48

## Data Availability

The original contributions presented in this study are included in the article. Further inquiries can be directed to the corresponding author.
